# Electroacupuncture Reduces Postoperative Pain and Analgesic Consumption in Patients Undergoing Thoracic Surgery: A Randomized Study

**DOI:** 10.1155/2016/2126416

**Published:** 2016-03-17

**Authors:** Tongyu Chen, Ke Wang, Jianjun Xu, Wen Ma, Jia Zhou

**Affiliations:** ^1^Department of Cardiothoracic Surgery, Shuguang Hospital Affiliated to the Shanghai University of Traditional Chinese Medicine, Shanghai 201203, China; ^2^Department of Acu-Moxibustion, Shuguang Hospital Affiliated to the Shanghai University of Traditional Chinese Medicine, Shanghai 201203, China

## Abstract

The aim of this study was to evaluate the effect of electroacupuncture (EA) on postoperative pain management in patients undergoing thoracic surgery. A randomized study was conducted. Ninety-two thoracic surgical patients were randomly divided into an EA group and a sham group. Postoperative intravenous analgesia was applied with a half dose of the conventional drug concentration in both groups. In the EA group, EA treatment was administered for three consecutive days after the surgery with 6 sessions of 30 min each. Compared with the sham group, patients in the EA group had a lower visual analogue scale (VAS) score at 2, 24, 48, and 72 hours and consumed less analgesic after surgery. The incidence of opioid-related adverse effects of nausea was lower in the EA group. The time to first flatus and defecation was also shorter in the EA group. Furthermore, the plasma *β*-endorphin (*β*-EP) level was higher by radioimmunoassay and the plasma 5-hydroxytryptamine (5-HT) level was lower in the EA group by enzyme-linked immunosorbent assay during the first 72 hr after thoracic surgery. Therefore, EA is suitable as an adjunct treatment for postoperative pain management after thoracic surgery.

## 1. Introduction

Thoracotomy can cause significant postoperative pain especially at the first 48 hours after the surgery [1]. Uncontrolled postthoracotomy pain is an independent risk factor for the development of postoperative complications such as fever and respiratory failure [2]. Poorly controlled postthoracotomy pain has led to the development of chronic thoracic syndrome and has a significant negative impact on the quality of life of these patients [3]. Various pain interventions including intravenous analgesia and epidural and peripheral nerve blocks have been used as part of multimodal pain management in caring for patients undergoing thoracotomy [4]. However, despite these well-established pain controlling methods, postthoracotomy pain remains.

Acupuncture has been used for thousands of years for various medical conditions including pain management. Electroacupuncture (EA) is one of the needle stimulation techniques that utilize electricity instead of manual manipulation. EA analgesia has been found to be effective as adjunctive therapy for postoperative pain management in patients that underwent total knee arthroplasty, prostatectomy, and thyroid surgery [5–7]. The use of EA has decreased the pain intensity, the consumption of cumulative opioids, and the side effect associated with the use of opioids. To date, only one prior study utilized EA as part of multimodal pain management for patients undergoing thoracotomy [8]. However, the sample size is too small (only 27 patients) to achieve statistical significant differences. Previous clinical and animal studies have demonstrated that endogenous opioids play a pivotal role in EA analgesia. Low-frequency (2 Hz) EA is highly effective in triggering the release of *β*-endorphin (*β*-EP) [9]. EA was also found to affect the release of 5-hydroxytryptamine (5-HT) [10]. Therefore, we designed this clinical trial not only to evaluate whether EA can serve as an adjunct to intravenous analgesic but also to evaluate whether it affects the endogenous *β*-EP and 5-HT release for patients undergoing small incision thoracotomy. Our primary aim is to determine whether EA can reduce postoperative analgesic requirement over the first 72 hours after surgery, and the secondary aim is to determine whether the incidence of opioid-related side effect (i.e., PONV) is less in EA treatment group than in the sham control group. The blood sample will be collected at 2 hr, 24 hr, 48 hr, and 72 hr after surgery.

## 2. Materials and Methods

### 2.1. Research Design and Participants

This study was conducted in Shuguang Hospital Affiliated to Shanghai University of Traditional Chinese Medicine from January 2012 to July 2014. Ninety-two patients were assigned to either an EA group (*n* = 46) or a sham group (*n* = 46) using a randomization sequence based on a table of randomly generated numbers. To conceal the allocation, randomization was carried out using sequentially numbered, opaque, sealed envelopes. The trial was approved by the Ethics Committee of the Shuguang Hospital Affiliated to Shanghai University of Traditional Chinese Medicine. All participating patients gave signed informed consent before being enrolled in this study.

**Figure 1:**
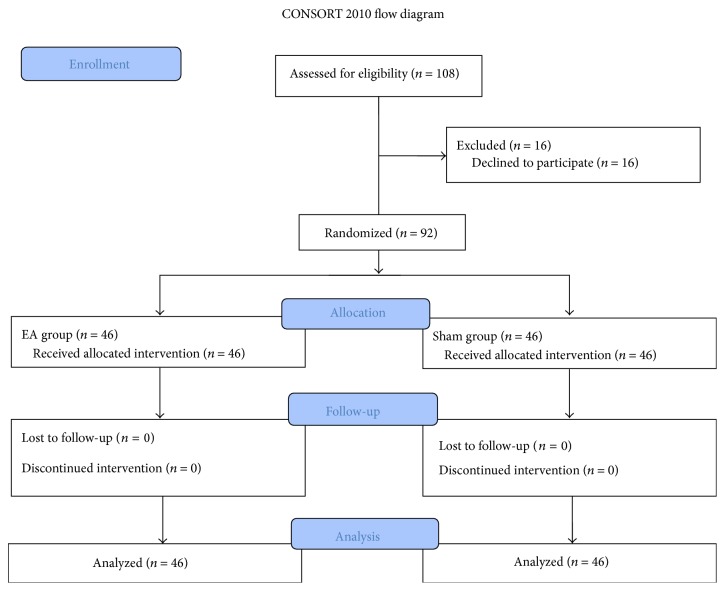
Study flow diagram.

Patients undergoing small incision lobectomy surgery participated in the study. Inclusion criteria were as follows: age 16–80 years, general anesthesia, and American Society of Anesthesiologists (ASA) grade I-II. Exclusion criteria were as follows: use of antiemetics or morphine before surgery, usage of a pacemaker, history of arrhythmia or epilepsy, opiate dependence, abnormally shaped cutaneous lesions at the application sites, and previous participation in similar experiments (any acupuncture or EA treatment).  Figure 1 shows the flow chart of the research protocol and allocation of participants in this study.

### 2.2. Interventions

The acupuncture treatments were performed by an acupuncturist with 10 years of acupuncture experience. Patients in the EA group received postoperative EA starting 1 hour after surgery. This treatment was repeated every 12 hours for three days after surgery. Patients received a total of six EA treatments. The acupuncture needles (0.25 × 40 mm, Huatuo, Suzhou Medical Instruments Factory, Suzhou, China) were inserted into acupoints, including bilateral Taichong (LR 3), Yang Lingquan (GB 34), Waiguan (TE 5), and Chize (LU 5), which were selected based on traditional Chinese medicine (TCM) meridian theory. The depth of needle insertion was usually 6–10 mm based on the patient's skin thickness and subcutaneous fatty tissues at the site of the acupuncture points. Next, the performer manipulated the needles until the patient felt a* de qi* sensation (deep cramp-like sensation). EA stimulation was applied for 30 min using LH202H HANS acupuncture point nerve stimulator (Beijing Huawei Co. Ltd., Beijing, China). The stimulation was used at a current of 3–5 mA, depending on the subject's tolerance, with a low frequency (2 Hz). In the sham group, the same acupuncture needles that were used in the EA group were placed at similar acupoints and were secured by adhesive tape without penetrating the skin [11]. The electrodes were also connected but no electronic current was applied.

In our hospital, intravenous analgesia is generally used for postoperative pain management. The solution, which consists of 500 *μ*g of Fentanyl and 50 mg of Flurbiprofen, was diluted with normal saline to 100 mL and was intravenously injected at a speed of 2 mL/h. In this study, the half dose of the conventional drug concentration, 250 *μ*g of Fentanyl and 25 mg of Flurbiprofen, was used. If the visual analogue scale (VAS) scores exceeded four, despite the use of intravenous analgesia, an additional 1 mg/kg of sauteralgyl was injected intramuscularly.

### 2.3. Measurement

Pain intensity was assessed by a VAS score ranging from 0 (no pain) to 10 (extreme pain). A research assistant who was not involved in the patients' EA application performed the assessments at standard time points (1 hr, 2 hr, 24 hr, 48 hr, and 72 hr after surgery). In addition to the VAS assessment, postoperative use of sauteralgyl for the first 72 hours was also recorded (mg/kg) along with other postoperative recovery data, such as the incidence of nausea and vomiting and the postoperative appearance of flatus and defecation.

The blood levels of *β*-EP and 5-HT were measured. Peripheral venous blood samples were collected into precooled anticoagulant tubes containing trasylol at 2 hr, 24 hr, 48 hr, and 72 hr after surgery. The plasma was separated into polyethylene tubes and was stored at −80°C until analysis. A radioimmunoassay was used to determine the plasma concentrations of *β*-EP. 5-HT was measured by using a commercial enzyme-linked immunosorbent assay (ELISA) kit (Labor Diagnostika Nord, Nordhorn, Germany) according to the manufacturer's instructions.

### 2.4. Data Analysis

Based on a small pilot study (six patients per group), we performed a power analysis to determine the sample size that was required to obtain significant effects for each VAS score at 24 h following surgery. We calculated that 39 patients per group would be sufficient (using power = 90% and type I error = 5) and allocated 45 per group to anticipate withdrawals. Chi-squared test was used for the comparison of proportions. One-way repeated ANOVA followed by Bonferroni's multiple comparison test was used to compare the values between the groups at each time point. *t*-test was applied for the comparison of other continuous measurements where appropriate. All statistical analyses were performed using SPSS software version 16.0 (SPSS Inc., Chicago, IL, USA). The level of significance for all statistical tests was set at 0.05.

## 3. Results

Of the 108 patients who were eligible, 92 signed the informed consent and were included in the study (Figure 1). There was no significant difference between the EA group and the sham control group with regard to baseline demographic characteristics (*p* > 0.05, Table 1).

**Figure 2:**
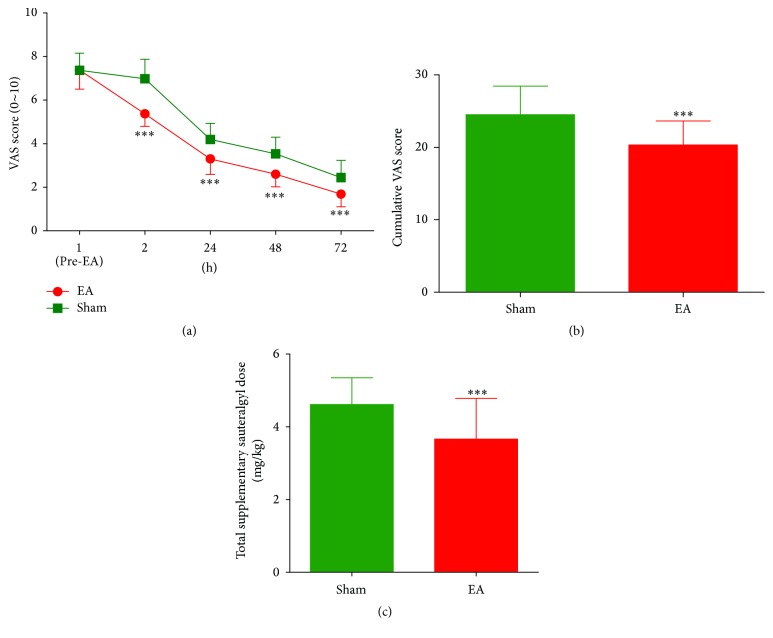
Postoperative visual analogue scale (VAS) score (a), cumulative VAS scores (b), and the supplementary sauteralgyl consumption (c) in either the electroacupuncture (EA) group (*n* = 46) or the sham control group (*n* = 46). ^*∗∗∗*^ *p* < 0.001* versus* sham group.

Figure 2(a) showed the pain VAS score over time for the two groups. Before EA stimulation, the VAS scores at 1 h were not significantly different between the two groups. However, significant differences were observed at 2 hr, 24 hr, 48 hr, and 72 hr after surgery between the EA group and the sham group (*F*_(1,360)_ = 30.54, *p* < 0.001). The time effect was significant in 2 hr, 24 hr, 48 hr, and 72 hr, compared to pre-EA stimulation (*F*_(4,360)_ = 345.31, *p* < 0.001). The interaction effect (time × group) was also significant (*F*_(90,360)_ = 118.23, *p* < 0.001), indicating time-dependent EA effects. The cumulative VAS showed significantly lower scores in the EA group compared with the sham group during the test period (*p* < 0.001, Figure 2(b)). The total dose of supplementary sauteralgyl was 9.2 ± 2.8 mg/kg in the EA group and 11.5 ± 1.8 mg/kg in the sham group (*p* < 0.0001), resulting in a 20% reduction in the EA group (Figure 2(c)). Additionally, nausea was more frequent in the sham group than in the EA group (*p* < 0.01, Table 2). However, postoperative vomiting did not significantly differ between the groups (*p* > 0.05, Table 2). The time of postoperative flatus and defecation was also shorter in the EA group (*p* < 0.0001, Table 2).

**Figure 3:**
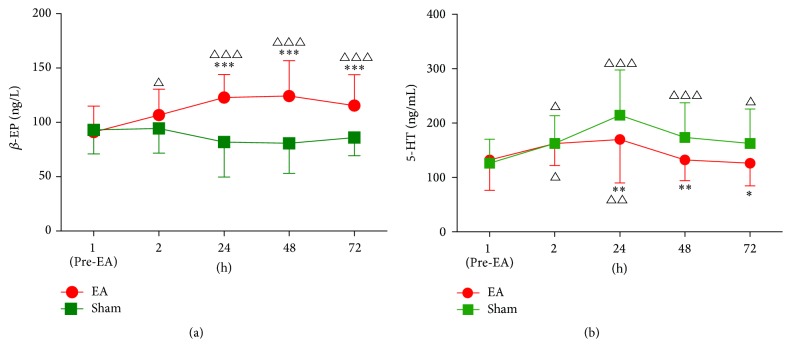
Plasma *β*-EP (a) and 5-HT (b) levels in patients receiving either the electroacupuncture (EA) treatment (*n* = 46) or the sham treatment (*n* = 46). ^△^ *p* < 0.05, ^△△^ *p* < 0.01, and ^△△△^ *p* < 0.001* versus* baseline; ^*∗*^ *p* < 0.05, ^*∗∗*^ *p* < 0.01, and ^*∗∗∗*^ *p* < 0.001* versus* sham group.

There were no significant differences in the *β*-EP and 5-HT levels between the groups at 1 hr after sugary. The *β*-EP level increased significantly at 2 hr, 24 hr, 48 hr, and 72 hr postoperatively in the EA group (*p* < 0.05 or *p* < 0.001), whereas the *β*-EP level decreased slightly in the sham group, resulting in a significant difference between the two groups at 24 hr, 48 hr, and 72 hr postoperatively (*F*_(1,360)_ = 104.59, *p* < 0.001) (Figure 3(a)). There was a sustained increase in plasma 5-HT levels postoperatively up to 72 h in the sham group (*p* < 0.05 or *p* < 0.001) (Figure 3(b)). However, compared with the sham group, the upward trend of 5-HT levels was suppressed in the EA group at 24 hr, 48 hr, and 72 hr postoperatively (*F*_(1,360)_ = 9.79, *p* < 0.01) (Figure 3(b)).

## 4. Discussion

Acupuncture anesthesia or acupuncture anesthesia combined with narcotic drugs has been widely reported in various types of surgeries, for example, in open heart surgery, pneumonectomy, hip replacement, hysteroscopic surgery, and nasal endoscopic sinus surgery [12–16]. These previous reports have already confirmed that perioperative EA is safe and effective in reducing perioperative analgesic requirements and postoperative morbidity [12–16].

Postthoracotomy pain is a significant issue and still remains a challenge to thoracic surgeons [17]. Many anesthetic and pharmacological agents are used to decrease postthoracotomy pain. However, these drugs carry risks of side effects and control the postthoracotomy pain effectively [18]. Furthermore, a literature search suggests that acupuncture analgesia is more efficient in surgical patients when administered postoperatively [19]. Therefore, our study aimed to determine whether postoperative EA can alleviate postoperative pain and reduce analgesic consumption and its related adverse effects.

In this study, our results indicate that postoperative EA reduced postoperative pain in thoracotomy patients compared with the sham group. Following the reduction of postthoracotomy pain, EA also decreased overall analgesic consumption by 20% in the first 72 hr postoperatively. Our results are similar to those of recent clinical trials where EA can be an effective adjunct for postsurgical pain management [20–22]. Furthermore, a systematic review found that acupuncture treatment reduced opioid consumption by 21–29% in fifteen RCTs and had clinical significance [23]. In contrast, one study showed that using implantation of small intradermal needles at acupoints did not reduce pain or analgesic requirements after thoracotomy when compared with a sham technique [24]. There could be two explanations for this controversy. First, we focused on acute postthoracotomy pain within 72 hours. However, the other study focus was on chronic postthoracotomy pain. Second, preoperative implantation of small intradermal needles was used, and the needles were retained for 4 weeks in the study [24]. This special acupuncture technique may not have delivered sufficiently strong stimulation to produce an analgesic effect [25, 26]. At present, there is a general consensus that the analgesic effect of acupuncture most likely resulted from activation of the endogenous pathway by stimulating the release of central endogenous opioid peptides *β*-EP and endomorphin, especially with low-frequency stimulation [27, 28]. The result of our study also supports the notion that 2 Hz EA is able to increase the plasma *β*-EP concentration and in turn that led to the reduction of acute postthoracotomy pain.

Nausea and vomiting are the most common side effects of opioid use. Although the frequency of postoperative vomiting was found to be similar between the two groups, the incidence of nausea in the EA group was significantly lower than that in the sham group during the 72 hr postoperative period. This observation may be caused by the fact that patients in the EA group have required less amount of postoperative analgesia than the sham group and/or the lower plasma level of 5-HT in EA group than in sham group. As 5-HT is one of the key modulators that regulate the motility of gastrointestinal tract, EA may regulate the release of 5-HT and resulted in faster returning of bowel function [29], as demonstrated in our results shortening the time to first flatus and defecation. This may also be the less analgesic consumption, hence less chance for the incidence of nausea.

Although there are different central mechanisms between acupoints and nonacupoints, the penetration of a needle through the skin can produce a physiological effect [30]. Furthermore, even with the same stimulus mode, different acupoints have different responses in the brain and different therapeutic effects [31, 32]. To exclude the placebo effect, the sham group used similar acupoints in this study. In some cases, however, there was no obvious placebo effect [20]. There are several limitations in our current study. First, we did not include a “control” group in our study design. However, a previous study suggested that the sham group is not superior to the control group [20]. Second, we did not record pain score during activities.

In conclusion, postoperative EA is advantageous in reducing acute postoperative pain in the first 72 hours postoperatively, analgesic consumption, incidence of nausea, and the time to first flatus and defecation. Thus, EA is an effective adjunctive therapy for postoperative pain management in patients undergoing thoracotomy. Future studies will focus on whether postoperative EA improves patient's recovery and prevents the development of chronic thoracotomy pain syndrome.

## Figures and Tables

**Table 1:** Demographic data.

Variables	EA group	Sham group	*p* value
Age (mean ± SD)	55.80 ± 15.75	56.93 ± 14.61	0.73
Gender (*n*, %)			
Male	32 (69.6)	36 (78.3)	0.34 (*χ* ^2^ = 0.90)
Female	14 (30.4)	10 (21.7)	
Weight (kg)	63.02 ± 7.98	60.50 ± 7.84	0.18

Values are number of patients (percentages).

**Table 2:** Incidence of nausea and vomiting.

Symptoms	EA group (*n* = 46)	Sham group (*n* = 46)	*p* value
Nausea	10 (21.7%)	22 (47.8%)	<0.01 (*χ* ^2^ = 6.90)
Vomiting	3 (6.5%)	5 (10.9%)	0.459 (*χ* ^2^ = 0.55)
Flatus (h)	24.3 ± 8.2	35.7 ± 7.76	<0.001
Defecation (h)	42.7 ± 13.9	59.2 ± 11.3	<0.001

Values are number of patients (percentages).
